# DNA plasmid coding for *Phlebotomus sergenti* salivary protein PsSP9, a member of the SP15 family of proteins, protects against *Leishmania tropica*

**DOI:** 10.1371/journal.pntd.0007067

**Published:** 2019-01-11

**Authors:** Elham Gholami, Fabiano Oliveira, Tahereh Taheri, Negar Seyed, Safoora Gharibzadeh, Nasim Gholami, Amir Mizbani, Fatemeh Zali, Sima Habibzadeh, Daniel Omid Bakhadj, Claudio Meneses, Kambiz Kamyab-Hesari, Alireza Sadeghipour, Yasaman Taslimi, Fatemeh khadir, Shaden Kamhawi, Mohammad Ali Mazlomi, Jesus G. Valenzuela, Sima Rafati

**Affiliations:** 1 Department of Medical Biotechnology, School of Advanced Technologies in Medicine, Tehran University of Medical Sciences, Tehran, Iran; 2 Department of Immunotherapy and *Leishmania* Vaccine Research, Pasteur Institute of Iran, Tehran, Iran; 3 Vector Molecular Biology Section, Laboratory of Malaria and Vector Research, National Institute of Allergy and Infectious Diseases, National Institutes of Health, Rockville, Maryland, United States of America; 4 Department of Epidemiology and Biostatistics, Pasteur Institute of Iran, Tehran, Iran; 5 Research Centre for Emerging and Reemerging Infectious Disease, Pasteur Institute of Iran, Tehran, Iran; 6 Department of Health Sciences and Technology, ETH Zurich, Zurich, Switzerland; 7 Department of Dermatopathology, Razi Hospital, Tehran University of Medical Sciences, Tehran, Iran; 8 Department of Pathology, Hazrat-e-Rasoul Akram Hospital, Iran University of Medical Sciences, Tehran, Iran; University of Notre Dame, UNITED STATES

## Abstract

**Background:**

The vector-borne disease leishmaniasis is transmitted to humans by infected female sand flies, which transmits *Leishmania* parasites together with saliva during blood feeding. In Iran, cutaneous leishmaniasis (CL) is caused by *Leishmania (L*.*) major* and *L*. *tropica*, and their main vectors are *Phlebotomus (Ph*.*) papatasi* and *Ph*. *sergenti*, respectively. Previous studies have demonstrated that mice immunized with the salivary gland homogenate (SGH) of *Ph*. *papatasi* or subjected to bites from uninfected sand flies are protected against *L*. *major* infection.

**Methods and results:**

In this work we tested the immune response in BALB/c mice to 14 different plasmids coding for the most abundant salivary proteins of *Ph*. *sergenti*. The plasmid coding for the salivary protein *PsSP9* induced a DTH response in the presence of a significant increase of IFN-γ expression in draining lymph nodes (dLN) as compared to control plasmid and no detectable PsSP9 antibody response. Animals immunized with whole *Ph*. *sergenti* SGH developed only a saliva-specific antibody response and no DTH response. Mice immunized with whole *Ph*. *sergenti* saliva and challenged intradermally with *L*. *tropica* plus *Ph*. *sergenti* SGH in their ears, exhibited no protective effect. In contrast, *PsSP9*-immunized mice showed protection against *L*. *tropica* infection resulting in a reduction in nodule size, disease burden and parasite burden compared to controls. Two months post infection, protection was associated with a significant increase in the ratio of IFN-γ to IL-5 expression in the dLN compared to controls.

**Conclusion:**

This study demonstrates that while immunity to the whole *Ph*. *sergenti* saliva does not induce a protective response against cutaneous leishmaniasis in BALB/c mice, *PsSP9*, a member of the *PpSP15* family of *Ph*. *sergenti* salivary proteins, provides protection against *L*. *tropica* infection. These results suggest that this family of proteins in *Ph*. *sergenti*, *Ph*. *duboscqi* and *Ph*. *papatasi* may have similar immunogenic and protective properties against different *Leishmania* species. Indeed, this anti-saliva immunity may act as an adjuvant to accelerate the cell-mediated immune response to co-administered *Leishmania* antigens, or even cause the activation of infected macrophages to remove parasites more efficiently. These findings highlight the idea of applying arthropod saliva components in vaccination approaches for diseases caused by vector-borne pathogens.

## Author summary

Leishmaniasis is a vector-borne disease transmitted to humans by an infected sand fly bite, through which *Leishmania* parasites and saliva are co-delivered into the host skin. Despite the numerous studies performed in this area, no vaccine is yet available to control this neglected disease in humans. During the past two decades, saliva of sand flies has been tested for possible application as a vaccine against leishmaniasis. Exposure to specific salivary proteins or sand fly bites can induce a protective cell-mediated immunity. Immunization with *Ph*. *papatasi* saliva or recombinant *PpSP15* has been previously reported to provide protection against *L*. *major* infection. In this study, the efficiency of immunization with *Ph*. *sergenti* saliva or plasmid coding for *Ph*. *sergenti* salivary proteins in protecting the BALB/c mice against *L*. *tropica* was explored. Here we show that although immunization with whole saliva induces a humoral response, this immune response is unable to protect mice against infection; in contrast, immunization with a plasmid coding for *Ph*. *sergenti PsSP9* salivary protein induces a Th1 immune response characterized by a strong DTH response, no detectable antibody response, and a high expression ratio of IFN-γ to IL-5 in lymph nodes. The Th1 induced immunity in *PsSP9*-immunized mice correlates with the protection observed against infection with *L*. *tropica*, which was associated with a significant reduction in ear thickness and decrease in parasite load in the ear of protected animals in comparison to control group. Our data suggest that instead of using the whole sand fly salivary proteins, a more effective approach is the use of a single *Ph*. *sergenti* salivary protein.

## Introduction

As a vector-borne parasitic and tropical disease, leishmaniasis has been reported in 98 countries (in 4 continents) with an annual incidence level of 0.9–1.6 million individuals, and 350 million individuals are at the risk of this infection [1,2]. The *Leishmania* parasite, as the causative agent of this disease, is delivered to the host during blood-feeding by infected female sand flies, namely, the *Phlebotomus* species in the Old World (Asia, Africa and Europe) and the *Lutzomyia* species in the New World (Central and South America). Various clinical symptoms, in visceral, mucocutaneus and cutaneous forms have been reported for leishmaniasis. The most commonly observed type of this disease is CL, which results in ulcers and permanent scars on the affected regions of the body and serious disabilities for the patient. Clinical forms of leishmaniasis are geographically distributed depending on the availability of the pathogenic species of *Leishmania* and their vectors. For example, CL has been mainly observed in Afghanistan, Brazil, Iran, Iraq and Syrian Arab Republic [1]. The fact that infection with certain species, including *L*. *major* or exposure to live *Leishmania* (leishmanization) results in long-term protection, in addition to the high costs of current treatments, drug toxicity and parasite resistance emergence intensify the need for producing a vaccine for this disease [3–5]. However, despite numerous investigations, no human vaccine for *Leishmania* is yet available [6].

Studies on development of *Leishmania* vaccines have focused on *Leishmania* antigens. In the past two decades, researchers started to examine sand fly salivary proteins [7], which are transmitted along with the parasite during blood feeding, as an alternative or a component of a vaccine against leishmaniasis. Different molecules are present in sand fly saliva which alter the hemostatic, inflammatory and immune response of the host, thereby facilitating parasite infection [8]. Simultaneous inoculation of *Leishmania* parasite and sand fly saliva has been reported to increase the parasite burden and cause exacerbation of the generated lesions [7,9–12]. However, immunization of animals with salivary components [13–15] or salivary gland homogenate (SGH) [12,16,17] from sand flies or exposing them to the bites of uninfected sand flies [18–25] resulted in protection against infection with *Leishmania* [26]. A cell-mediated immune response (CIR), as a form of a DTH response to sand fly salivary proteins, was shown to provide protection against *Leishmania* infection. This anti-salivary immunity appears to be important, considering that the parasite is unavoidably naturally injected along with salivary proteins into the biting site. Hence, similar to an adjuvant, a Th1 anti-saliva immunity can accelerate the induction of a protective Th1 immunity against *Leishmania* [27].

In Iran, zoonotic CL is caused by *L*. *major*, which is usually transmitted by the *Ph*. *papatasi* vector. On the other hand, anthroponotic CL is caused by *L*. *tropica* mainly transmitted by the *Ph*. *sergenti* sand fly [28]. According to the literature, exposing mice to the bites of uninfected sand flies or immunizing them with *Ph*. *papatasi* SGH can protect them against *L*. *major* infection caused by needle inoculation [7] or the bites of infected sand flies [13]. Immunization with *PpSP15*, a salivary protein from *Ph*. *papatasi*, provides protection in mice against *L*. *major* infection [20,21,29,30]. The immunity induced by *Ph*. *sergenti* salivary proteins and their effects on *L*. *tropica* infection have not been previously reported. Therefore, in this work, the immunogenicity of *Ph*. *sergenti* salivary components were assessed and we tested whether immunizing BALB/c mice with *Ph*. *sergenti* SGH or DNA plasmids coding for *Ph*. *sergenti* salivary proteins can lead to an appropriate immunity to control *L*. *tropica* infection.

## Materials and methods

### Chemicals, media, reagents and instruments

Apyrogenic deionized water (Milli-Q System, Millipore, Molshem, France) was used to prepare all needed solutions. The Endo-Free Plasmid Mega kit, RNeasy Mini Kit, Quanti Nova SYBR Green Master Mix and Anti-His antibody were purchased from QIAGEN, Germany. TRIzol Reagent and SuperScript III First-Strand Synthesis system were from Thermo Fisher Scientific (Invitrogen Company, USA). The materials needed for PCR reaction, agarose gel electrophoresis and enzymatic digestion were all provided by Roche Applied Sciences, Germany. Diaminobenzidine (DAB) powder, Bovine Serum Albumin (BSA) and acrylamide were provided by Merck, Germany. Horseradish peroxidase-conjugated goat anti-mouse IgG, Urea, Ponceau-S, Sodium dodecyl sulfate (SDS), Tris-base, Tris-HCL, M199 medium, RPMI-1640, DMEM, gentamicin, kanamycin, L-glutamine, hemin, HEPES, adenosine and Ficoll-400 were purchased from Sigma, Germany. Fetal Calf Serum (FCS) and Schneider insect media were provided by Gibco (Life Technologies, Germany). Goat anti-mouse IgG1-HPR and IgG2-HPR were obtained from Southern Biotech, Canada. Peroxidase Substrate System as an ELISA substrate was from KPL (ABTS, USA). Linear Polyethylenimine was purchased from Polyscience, Germany. Protran Nitrocellulose Transfer Membranes was from Schleicher & Schuell BioScience, Germany. GF-1 Tissue DNA Extraction Kit was purchased from Vivantis Technologies, Malaysia. The Agilent RNA 6000 Nano reagent kit were purchased from Agilent Technologies, USA. The Agilent 2100 Bioanalyzer instrument (Agilent Technologies, USA), ELISA reader (Tecan, USA), NanoDrop (Nanodrop, ND-1000, USA) and Speed Vac (Thermo Scientific, USA) devices were also used in this investigation.

### Salivary gland homogenate (SGH) preparation

*Ph*. *sergenti* were kept in the insectary at the Laboratory of Malaria and Vector Research, National Institutes of Health, NIH (Rockville, MD, USA). The salivary glands were dissected from 5- to 7-day-old and non-blood fed female sand flies and then transferred to PBS for subsequent storage at −70 °C. After disruption by ultra-sonication and centrifugation, the produced supernatant was collected and dried in a Speed Vac device and reconstituted before use.

### Ethical statement

Female BALB/c mice, (6–8 weeks old) with weight range of 18–20 g were obtained from Pasteur Institute of Iran. The animals under investigation were maintained, handled, anesthetized and euthanized under the approval of Institutional Animal Care and Research Advisory Committee of Pasteur Institute of Iran (ethical code: IR.RII.REC.1394.0201.6417, dated 2015). All experiments were designed and carried out according to the Specific National Ethical Guidelines for Biochemical Research (2005) by the Research and Technology Deputy of Ministry of Health and Medicinal Education (MOHM) of Iran. Mice were euthanized through cervical dislocation method. Animals were anesthetized via intraperitoneal (i.p.) administration of Xylazine/Ketamine anesthetizing cocktail [31] in order to minimize suffering animals under investigation.

### Preparation of plasmids encoding *Ph*. *sergenti* salivary proteins

The mammalian codon optimized nucleotides encoding the N-terminus to the stop codon of the most abundant secreted proteins from *Ph*. *sergenti* salivary gland proteins were cloned in a modified mammalian expression plasmid (VR1020-TOPO) through T/A cloning strategy and topoisomerase technology and then transformed into the DH5α strain. The VR1020-TOPO plasmid has features such as a CMV promoter, the signal-secretory peptide of tissue plasminogen activator (TPA), replacing the sand fly specific-secretory signal peptide and a 6×His-tag, downstream from the target insert. This modified vector enables efficient production of the secreted proteins in animal tissues and other mammalian-based expression systems. The fourteen cloned transcripts used in this study are *PsSP7* (HM560864, D7-related proteins), *PsSP9* (HM569364, PpSP15-like protein), *PsSP14* (HM560870, PpSP15-like protein), *PsSP15* (HM560868, PpSP15-like protein), *PsSP20* (HM560866, yellow-related proteins), *PsSP26* (HM569362, yellow-related proteins), *PsSP40* (HM560860, apyrase), *PsSP41* (HM560862, apyrase), *PsSP42* (HM560861, apyrase), *PsSP44* (HM569368, PpSP32-like protein), *PsSP52* (HM537134, antigen 5-related proteins), *PsSP54* (HM569365, PpSP15-like protein), *PsSP73* (HM569367, unknown), and *PsSP98* (HM569366, unknown). In order to purify all recombinant plasmids and the empty control plasmid (VR1020), an Endo-Free Plasmid Mega kit was used according to the manufacturer’s protocol.

### COS-7 cell transfection and confirmation of protein expression using western blot analysis

In order to confirm the expression of the 14 plasmids harboring each *Ph*. *sergenti* salivary protein, COS-7 cells (ATCC CRL-1651) were used as an expression host. Briefly, COS-7 cells were cultured in six-well plates (Greiner) in complete RPMI medium supplemented with 10% FCS at 37 °C in the presence of 5% CO_2_. For cell transfection, we used PEI/DNA complexes, prepared through mixing Linear Polyethylenimine (LINPEI, MW = 25 kDa, 10 μM) and 5 μg of each recombinant plasmid or VR1020 (as the control) [32]. Expression of *Ph*. *sergenti* salivary proteins in transfected COS-7 cells were confirmed by western blot analysis. In brief, forty eight hours after transfection, the supernatant of transfected COS-7 cells were harvested, then mixed with SDS-PAGE sample buffer and boiled for 5 min, and run on a 12.5% or 15% SDS-PAGE gel. Proteins were then transferred from gel to nitrocellulose membranes by electro-blotting. Free binding sites on nitrocellulose membrane were blocked with blocking solution (PBS with 0.05% Tween 20 and 2.5% BSA) for 2 hours. After three washes, the membrane was incubated with HRP-conjugated goat anti-mouse IgG (1:2000) for 2 hours at room temperature and visualized using the 3,30-diaminobenzidine substrate (DAB).

### Intradermal immunization with *Ph*. *sergenti* salivary DNA plasmids

In order to assess the immunogenic characteristics of DNA plasmids coding for *Ph*. *sergenti* salivary proteins, 6–8 weeks old BALB/c mice (6 mice in each experimental group) were i.d. immunized three times at two-week intervals using 30-gauge needle in the right ear. For this purpose, we used 10 μg of plasmid (either the empty plasmid control or a recombinant plasmid encoding a *Ph*. *sergenti* salivary protein), an equivalent of 0.5 *Ph*. *sergenti* salivary gland pair or PBS; all in a total volume of ~10 μl with PBS.

### Analysis of specific anti-saliva antibodies by ELISA

ELISA microplates were coated with 100 μl of SGH diluted to two pairs of SGHs/ml in coating buffer (Na_2_CO_3_ 0.02 M, NaHCO_3_ 0.45 M, pH 9.6) overnight at 4 °C. Following rinsing with PBS-0.05% Tween, wells were blocked using 100 μl of 1% BSA in PBS for 2 h at 37 °C. Wells were incubated for 3 h with sera from mice immunized with recombinant or control plasmids obtained two weeks after the last immunization and diluted (1:50) in PBS-0.05% Tween-1% BSA. After one more washing step, wells were incubated with Horseradish peroxidase-conjugate goat anti-mouse IgG diluted (1:5000) in PBS-0.05% Tween-1% BSA for 2 h at 37 °C. Following another washing step, plates were incubated with Peroxidase Substrate System (KPL) as the substrate for 30 min at 37 °C. After stopping the reactions with 1% SDS, the absorbance was measured at 405 nm using an ELISA reader. The cut off value was determined by measuring anti-saliva IgG of plasmid control mice group (mean + 3 SD).

### Analysis of DTH and histopathological assessment after immunization in the ear dermis

Two weeks after the last step of immunization, the animals were inoculated i.d. into the left ear dermis with *Ph*. *sergenti* SGH (0.5 salivary gland pair per mouse) using a 30-gauge needle. Forty eight hours later, we measured ear thickness (DTH response) using a digital caliper (with a resolution of 0.01 mm). For histopathological assessment at this time point, after fixing the dissected ears in 10% phosphate-buffered formalin, they were processed, embedded in paraffin, and then the 5-μm sections prepared by microtome were stained using hematoxylin and eosin (H & E) and analyzed using light microscopy. For morphometric analyses, inflammatory cells were counted in three fields/section using a 400× magnification, covering a total area of 710 mm^2^.

### Immunization with Th1 DTH-inducing *Ph*. *sergenti* salivary DNA plasmid and intradermal challenge with *L*. *tropica* parasites plus *Ph*. *sergenti* SGH

After screening the Th1 DTH response in BALB/c mice which was triggered by the saliva proteins of *Ph*. *Sergenti*, the animals (10 mice per group) were immunized three times with two-week intervals using the selected plasmid encoding *Ph*. *sergenti* salivary protein, SGH, empty plasmid or PBS in the right ear using a 30-gauge needle. Two weeks after the last immunization, animals were challenged i.d. into the left ear dermis with 10^7^ metacyclic *L*. *tropica* parasites plus *Ph*. *sergenti* SGH (0.5 salivary gland pair) using a 30-gauge needle in an almost 10 μl total volume. The *L*. *tropica* parasite named as MOHM/IR/09/Khamesipour-Mashhad was isolated from patient in city of Mashhad, Iran, in 2009 (provided as gift by Dr. Ali Khamesipour). *L*. *tropica* promastigotes were cultured in M199 medium supplemented with 10% hi-FCS. The Ficoll-400 step-gradient was used to isolate the *L*. *tropica* metacyclic promastigotes [33]. To mimic the natural model of infection and examine whether immunity to the salivary proteins of the sand fly can protect the animals against CL, mice were infected by injection of parasites together with *Ph*. *sergenti* SGH into their ear dermis. The ear thickness was monitored and measured weekly using a digital caliper. To measure the disease burden (area under the curves, AUC), the ear thickness of each individual immunized mouse was recorded once per week. A disease course curve for each mouse in the experimental and control groups was separately obtained. Prism (GraphPad Software) was used to calculate AUC.

### Analysis of cytokine expression profile by Real-time PCR

Cytokine profiles were analyzed at two time points: once after *Ph*. *sergenti* SGH inoculation (to screen the Th1 DTH response against each DNA plasmid), and once after infectious challenge with *L*. *tropica* plus *Ph*. *sergenti* SGH. For this purpose, total RNA was extracted from the mouse dLN using TRIzol reagent and then RNeasy Mini Kit. The quality of extracted RNA was confirmed using an Agilent RNA 6000 Nano reagent kit. For first-strand cDNA synthesis, approximately 2 μg of RNA reverse-transcribed in a total volume of 20 μl using SuperScript III reverse transcriptase according to the manufacturer’s instructions. For quantification of gene expression, the 1:10 diluted cDNA was subjected to the reaction containing 5 pmol of each forward and reverse primer and 12.5 μl Quanti Nova SYBR Green Master Mix in a 25 μl total volume. Real-time PCR reactions were performed in duplicates on an Applied Biosystems 7500 instrument. Thermal cycles with an initial incubation step at 95 °C for 5 min followed by 45 cycles at 95 °C for 10 s, at 60 °C for 15 s, and at 72 °C for 35 s. The mRNA levels of each target gene were normalized to that of HPRT. The results are shown in fold change compared to the PBS control. Gene expression was analyzed based on the comparative method. The cycle threshold (C_t_) values for cytokines were normalized to the expression of HPRT based on the following formulation: ΔC_t_ = C_t (target gene)_−C_t (HPRT gene)_. We obtained the fold change using 2^−ΔΔCt^, in which ΔΔC_t_ = ΔC_t (test)_−ΔC_t (control)_ [34]. We used the following primers for real-time PCR: HPRT (Forward: *5′-*GTCCCAGCGTCGTGATTAG*-3′*; Reverse: *5′-*GAGCAAGTCTTTCAGTCCTGTC*-3′*); IFN-γ (Forward: *5′-*TCTGAGACAATGAACGCTACAC*-3′*; Reverse: 5*′-*CTTCCACATCTATGCCACTTGAG*-3′*); IL-5 (Forward: *5′-*TGACAAGCAATGAGACGATGAG*-3′*; Reverse: *5′-*CTCCAATGCATAGCTGGTGA*-3′*).

### Quantification of parasite propagation in the infected ear

Quantification of the parasites in the infected ear of animals in different groups was performed using Real-time PCR at one and two months after challenge. After euthanizing animals in each group (5 mice per group) the genomic DNA was extracted from each infected ear using GF-1Tissue DNA Extraction Kit according to manufacturer’s instruction. DNA concentration was measured by a NanoDrop device. The following primer set was used to target a part of *L*. *tropica* kinetoplastid minicircle DNA: (KDNA1F: (5'-GGGTAGGGGCGTTCTGC-3') and KDNA1R (5'-TACACCAACCCCCAGTTTGC-3')) [35,36]. The absolute copy number corresponding to the target sequence was measured on an Applied Biosystems 7500 real time PCR system. Standard *L*. *tropica* genomic DNA was used in 10-fold dilution corresponding to 2×10^8^ to 2×10^1^ parasites for drawing the standard curve. To quantify the parasites in tissues, 50 ng of DNA was applied to a reaction containing 5 pmol of each of the forward and reverse primers and 12.5 μl of Quanti Nova SYBR Green Master Mix in a 25 μl total volume. The PCR program was as the following: at 95 °C for 5 min; 40 cycles at 95 °C for 10 s, at 60 °C for 15 s, and at 72 °C for 35 s. All reactions were performed in duplicate.

### Statistical analysis

We replicated each measurement twice and averaged the obtained results. Shapiro-Wilk test was used to check the distribution of DTH response, Antibody response, IFN-γ, IL-5 and ratio of IFN-γ to IL-5 mRNA expression in dLN as well as parasite burden and disease burden (area under the curves, AUC). Due to the non-normality of all data, non-parametric van der Waerden’s normal score test and Dunn’s multiple comparison tests were used to compare the distribution of these variables between experimental and control group.

To assess the effect of variables on ear thickness, we used Linear Mixed Models for repeated measured data. The significant interaction between time and group factors implied that the experimental groups were changing with time, with different manners. A statistically significant interaction effect may indicate that the overall patterns of differences at the level of main effects are not likely to be consistent across all groups. The *p* values below 0.05 were considered as significant. Statistical analyses were carried out using Stata (14.0) (StataCorp. 2015. Stata Statistical Software: Release 14. College Station, TX: StataCorp LP) and R 3.4.3 (R Core Team (2017). R: A language and environment for statistical computing. R Foundation for Statistical Computing, Vienna, Austria) using the Rfit, PMCMR, nparcomp and multcomp packages.

## Results

### Confirmation of protein expression of all DNA constructs in COS-7 cell

**Fig 1:**
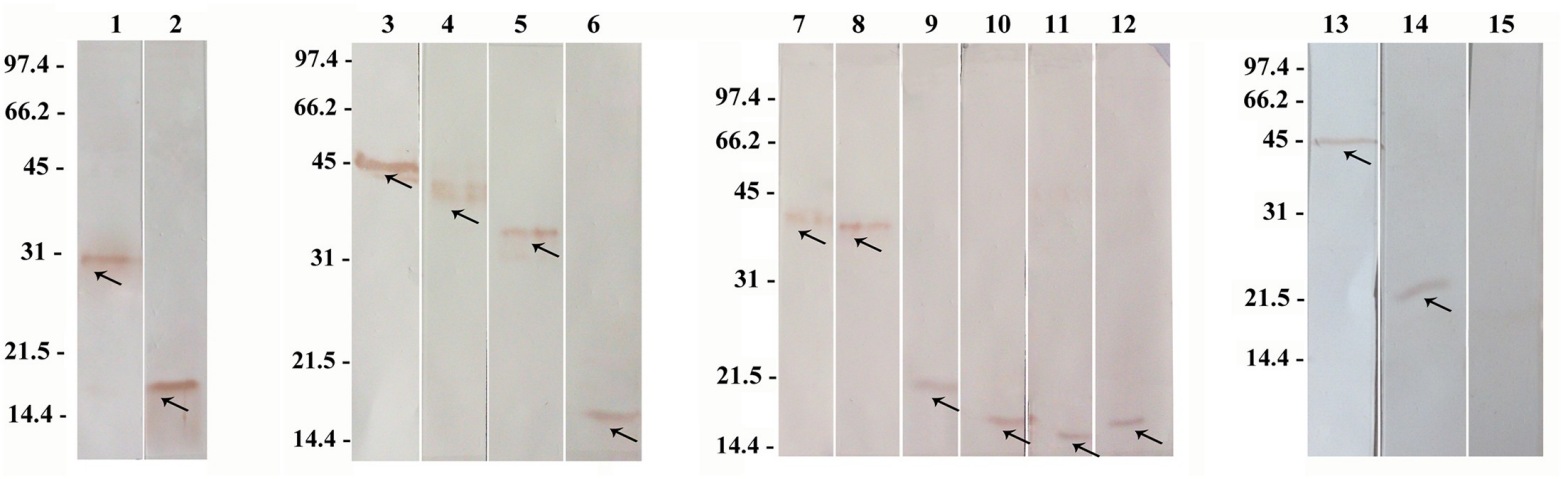
Confirmation of *Ph*. *sergenti* salivary proteins expression in supernatant of COS-7 cell culture using western blot analysis by anti-His antibody. COS-7 cell lines were transiently transfected with recombinant plasmids encoding for fourteen *Ph*. *sergenti* salivary proteins. Forty-eight h after transfection, the supernatants were subjected to western blot analysis using anti-His antibody. Line 1. VR1020-PsSP7 (MW, 30.4 KDa), line 2. VR1020-PsSP9 (17.8 KDa), line 3. VR1020-PsSP20 (46.1 KDa), line 4. VR1020-PsSP42 (39.6 KDa), line 5. VR1020-PsSP52 (32.5 KDa), line 6. VR1020-PsSP54 (18.4 KDa), line 7. VR1020-PsSP40 (39.3 KDa), line 8. VR1020-PsSP41 (37.4 KDa), line 9. VR1020-PsSP14 (20.8 KDa), line 10. VR1020-PsSP15 (18.4 KDa), line 11. VR1020-PsSP73 (16 KDa), line 12. VR1020-PsSP98 (18 KDa), line 13. VR1020-PsSP26 (46.2 KDa), line 14. VR1020-PsSP44 (24.8 KDa), line 15. VR1020.

Before screening the 14 different plasmids coding for *Ph*. *sergenti* salivary proteins in animals, we tested the protein expressions of these DNA constructs in COS-7 cells. The construct has a signal secretory peptide and we added to each transcript a histidine tag. We observed expression of protein in COS-7 cells from all plasmids (Fig 1).

### DNA encoding *PsSP9* of *Ph*. *sergenti* salivary protein induces a Th1 response in immunized mice

**Fig 2:**
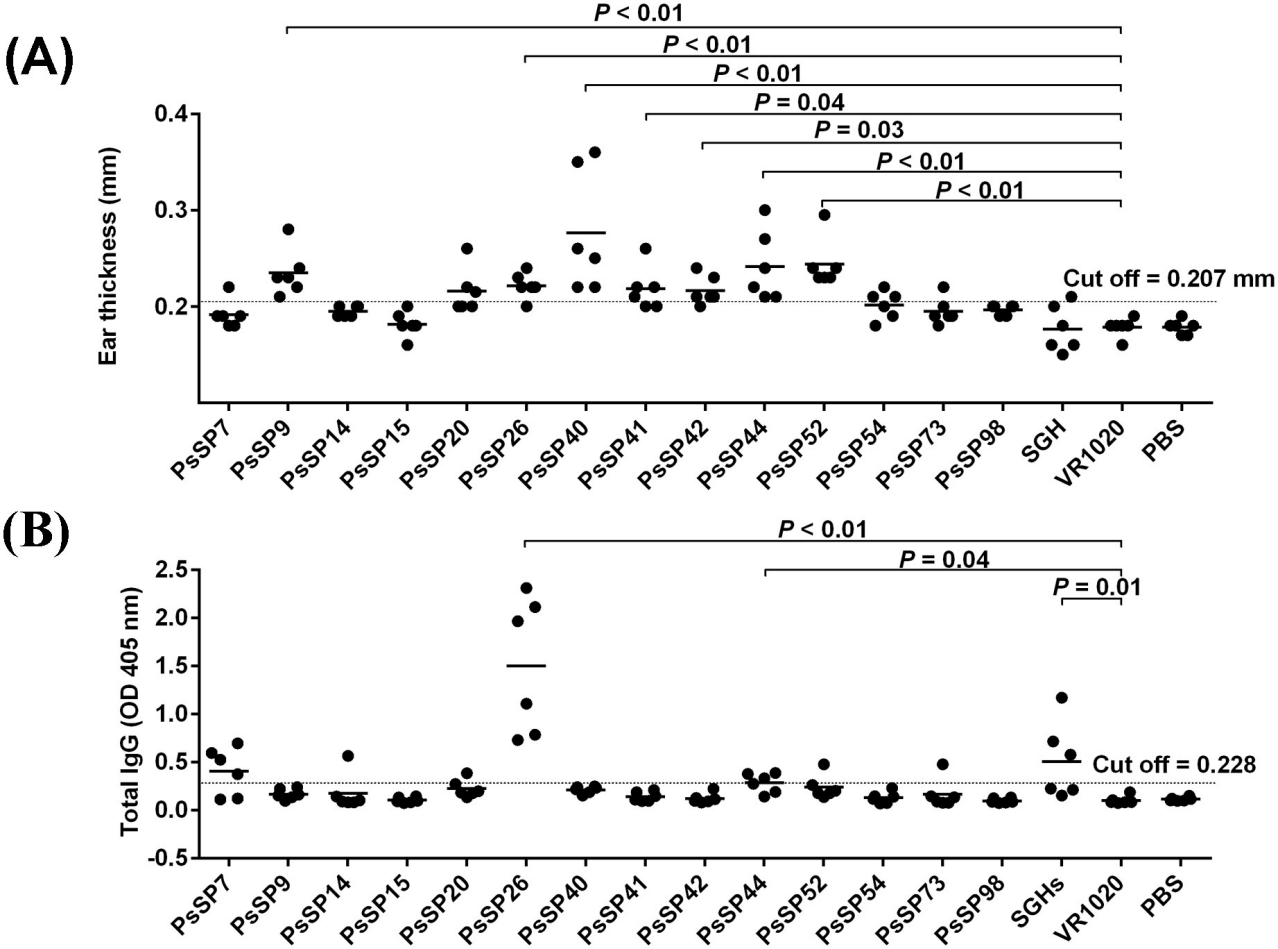
DTH and antibody responses to *Ph*. *sergenti* salivary proteins. BALB/c mice (six mice per group) were immunized in the right ear three times at two week intervals with plasmids encoding for fourteen *Ph*. *sergenti* salivary proteins (10μg), *Ph*. *sergenti* salivary gland homogenate (SGH, 0.5 pair), empty plasmid (VR1020, 10μg) or PBS (control) in total volume of 10 μl with PBS. Two weeks after the last immunization, the contralateral ear was intradermal challenged with 0.5 pairs of *Ph*. *sergenti* SGH. Data shown are from one representative of two independent experiments. (A) Delayed-type hypersensitivity response (DTH) generated at 48 h after *Ph*. *sergenti* SGH inoculation was determined. (B) Total IgG antibody response to *Ph*. *sergenti* SGH was determined by ELISA in immunized BALB/c. Statistical significance of the difference compared to the control empty-plasmid group (VR1020) is shown for each of the immunized groups. Dashed line, the cut off value determined as the mean + 3 SD of the response of the empty-plasmid control group.

The 14 different DNA plasmids coding for *Ph*. *sergenti* salivary proteins were screened to select a plasmid that can induce a Th1 cellular immune response. We also compared these plasmids to *Ph*. *sergenti* salivary gland homogenate. The parameters for selection were 1) The presence of a distinct DTH response associated with mononuclear cell infiltration in the ear and 2) increased levels of IFN-γ and low levels of IL-5 expression in dLN in comparison with the empty plasmid control group. Surprisingly, immunization with the whole *Ph*. *sergenti* salivary gland homogenate (SGH) did not induce a detectable DTH response (Fig 2A). In contrast, seven plasmids induced a positive DTH and showed greater ear thickness than the control plasmid that produced a median of 0.18 mm (Fig 2A). The median ranks of DTH responses were in the following descending order: *PsSP40* (0.26 mm), resulting in the greatest measurable response in animal skin; *PsSP52* (0.24 mm), *PsSP44* (0.23 mm), *PsSP9* (0.23 mm), *PsSP26* (0.22 mm), *PsSP41* (0.22 mm) and *PsSP42* (0.21 mm), as the smallest detectable levels. Groups immunized with *PsSP40* (*p* <0.01), *PsSP52* (*p* <0.01), *PsSP9* (*p* <0.01), *PsSP44* (*p* <0.01), *PsSP26* (*p* <0.01), *PsSP41* (*p* = 0.04) and *PsSP42* (*p* = 0.03) showed significantly higher DTH responses compared to the control plasmid (Fig 2A and S1 Table).

Specific total IgG antibody responses against *Ph*. *sergenti* SGH in the sera of all groups was measured. In comparison with the control groups (PBS and VR1020), the *Ph*. *sergenti* SGH-immunized mice group produced higher levels of anti-saliva-IgG than the cut off value (median at OD_405 nm_ = 0.40, S1 Table and Fig 2B). Moreover, the group immunized with *PsSP26*-encoding plasmid had the highest level of total IgG antibody production against *Ph*. *sergenti* SGH in comparison with the control plasmid group (median at OD_405 nm_ = 1.53, *p<0*.*01)*. In addition, the two other groups encoding the *PsSP7* and *PsSP44* proteins also produced higher levels of total IgG than the cut off value (*PsSP44* with median OD_405 nm_ = 0.30, *p* = 0.04 and *PsSP7* with median OD_405 nm_ = 0.45, *p* = 0.07) as demonstrated in Fig 2B and S1 Table.

**Fig 3:**
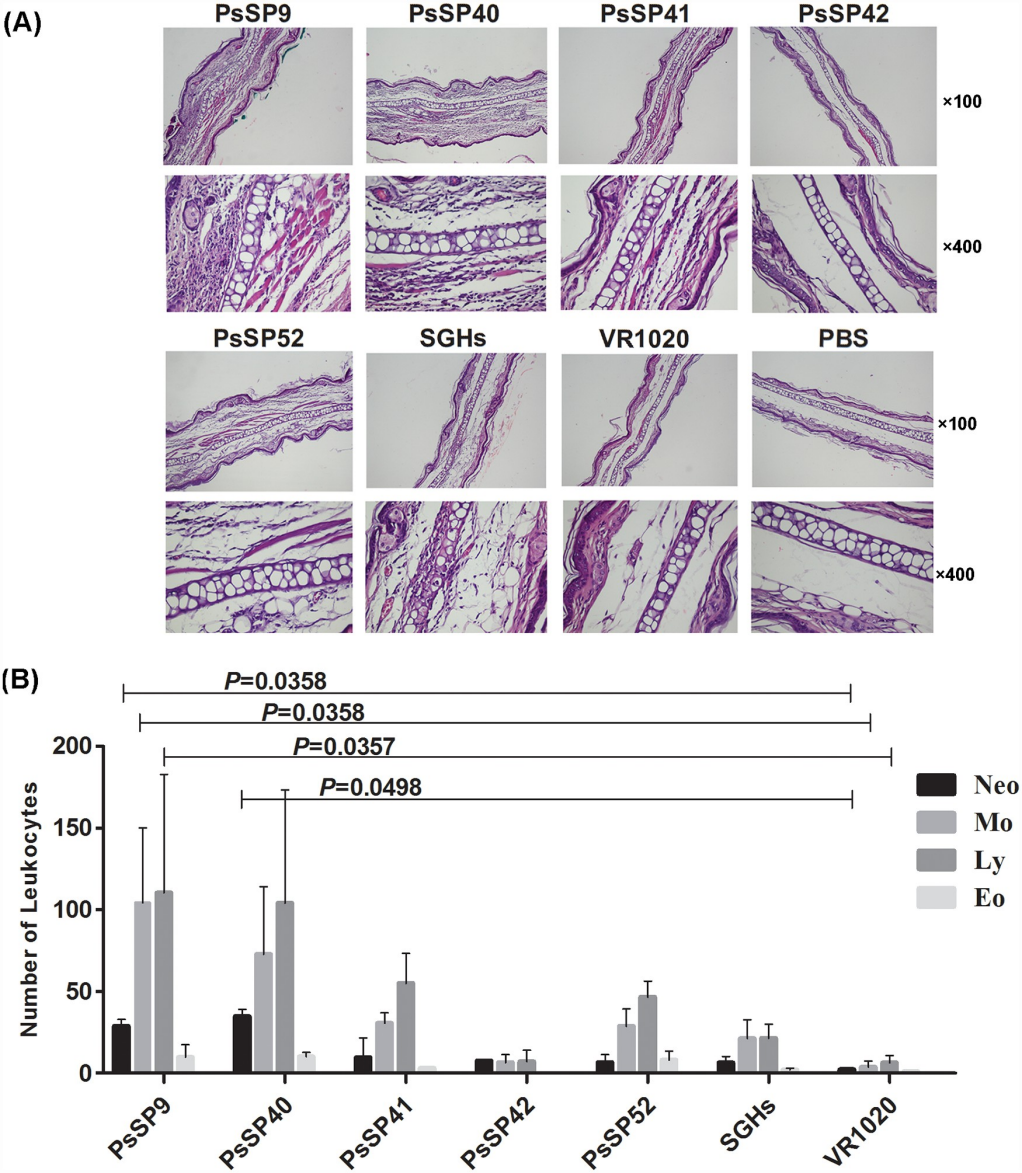
Histopathological analysis of the skin response to *Ph*. *sergenti* salivary proteins. Histological evaluation of representative ears of BALB/c mice immunized with *PsSP9*, *PsSP40*, *PsSP41*, *PsSP42*, *PsSP*52, *Ph*. *sergenti* SGH, empty plasmid, or PBS. Two weeks after the last immunization, mice were challenged in the opposite ear with *Ph*. *sergenti* salivary gland homogenate. Ear sections stained with hematoxylin and eosin (H&E staining). Sections were analyzed by optical microscopy under 100× and 400× (A) or 100× (B) magnification and the number of leukocytes enumerated microscopically. Bars represent the mean ±SD. Data are representative of two independent experiments (3–5 mice per group). The *p* value is indicated for each immunized group compared with the control plasmid group (VR1020).

Based on histological analysis 48 h after *Ph*. *sergenti* SGH injection in the ear dermis of plasmid-immunized animals, *PsSP9-* and *PsSP40-*immunized groups were characterized by a robust mononuclear infiltration mainly containing macrophages, lymphocytes and a lower number of neutrophils compared to the PBS- and plasmid- control groups (Fig 3). The number of inflammatory cells recruited in the *Ph*. *sergenti* SGH-immunized mice group was moderate and there were fewer cells than in the *PsSP9*- and *PsSP40-*immunized groups (Fig 3). Furthermore, the number of inflammatory cells recruited in *PsSP41- and PsSP52-*immunized mice was similar to that of *Ph*. *sergenti* SGH-immunized mice. No detectable increase in the number of inflammatory cells recruited in *PsSP42*-immunized mice was observed, compared to the PBS- and plasmid- control groups (Fig 3).

Based on the outcome of the DTH response, the antibody response (as shown in Table 1) and histological analysis against various salivary proteins, *PsSP9*, *PsSP40*, *PsSP41*, *PsSP42*, and *PsSP52* plasmids were selected for further evaluation of the cellular immune response in BALB/c mice.

**Table 1:** The immune response (DTH and antibody) induced in BALB/c mice by immunization with plasmids encoding for the most abundant secretory salivary proteins of *Ph*. *sergenti*.

Sequence name	Predicted molecular weight, kDa	NCBI accession no.	Protein annotation	DTH response	Antibody response
PsSP7	26.7	HM560864	D7-related	–	+
PsSP9	14	HM569364	PpSP15-like	+++	–
PsSP14	17.1	HM560870	PpSP15-like	–	–
psSP15	14.7	HM560868	PpSP15-like	–	–
PsSP20	42.4	HM560866	yellow-related	+	+
PsSP26	43.9	HM569362	yellow-related	+++	+++
PsSP40	35.6	HM560860	apyrase	+++	–
PsSP41	33.7	HM560862	apyrase	+++	–
PsSP42	35.9	HM560861	apyrase	+	–
PsSP44	22.5	HM569368	PpSP32-like	+++	+++
PsSP52	29	HM537134	antigen 5-related	+++	+
PsSP54	14.6	HM569365	PpSP15-like	–	–
PsSP73	12.2	HM569367	unknown	–	–
PsSP98	14.3	HM569366	unknown	–	–
SGH	-	-	-	–	+++
VR1020	-	-	-	–	–

SGH, *salivary gland homogenate*; VR1020, *empty plasmid*.

(−) no response detected; (+) weak response, (+++) strong response

**Fig 4:**
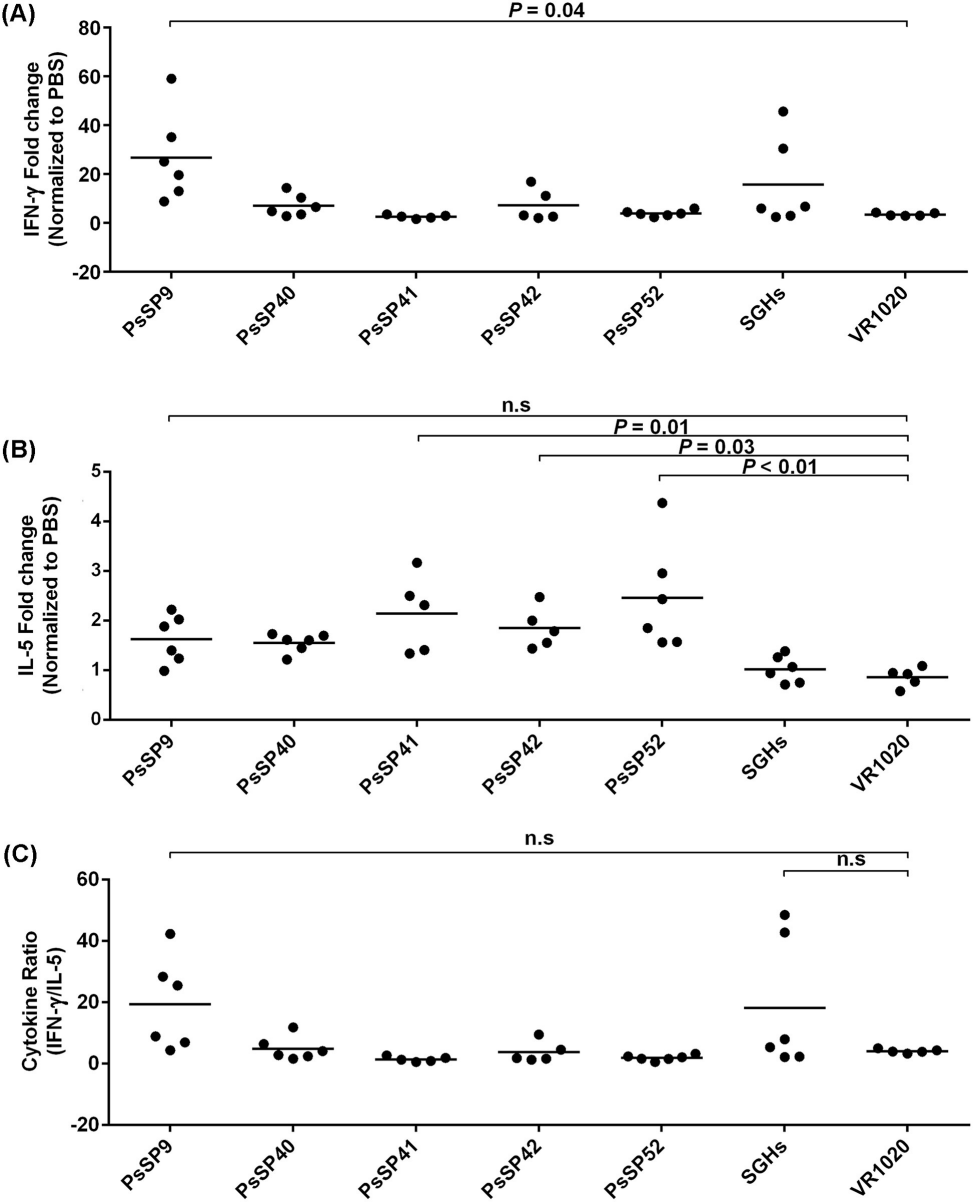
Cytokine profile expression in the dLN of mice immunized with selected plasmids 48 h after *Ph*. *sergenti* SGH injection. BALB/c mice (six mice per group) were intradermally injected in the right ear three times at two week intervals with plasmids encoding one of the five *Ph*. *sergenti* salivary proteins (10μg), *Ph*. *sergenti* salivary gland homogenate (SGH, 0.5 pair), empty plasmid (VR1020, 10μg) or PBS alone in total volume of 10 μl. Two weeks after the last immunization, the contralateral ear was intradermally challenged with 0.5 pairs of *Ph*. *sergenti* SGH. Data shown are from one representative of two independent experiments. (A) IFN-γ, (B) IL-5 and (C) the ratio of IFN-γ to IL-5 mRNA expression in the dLN of BALB/c mice immunized with *PsSP9*, *PsSP40*, *PsSP41*, *PsSP44*, *PsSP52*, empty plasmid, and PBS 48 h after challenge with *Ph*. *sergenti* SGH were analyzed. The obtained results were normalized to the expression level of HPRT. These data are presented as fold change relative to the PBS control. The *p* value is indicated for each immunized group compared to the control plasmid group (VR1020).

Forty-eight hours after *Ph*. *sergenti* SGH injection, mice immunized with the above-mentioned plasmids were euthanized and IFN-γ and IL-5 cytokine expression was evaluated in their dLN by Real-time PCR (Fig 4). *PsSP9* immunized mice was the only group that induced a statistically significant increase in IFN-γ mRNA expression, compared to the control plasmid group (median of fold change = 22.30, *p* = 0.04, Fig 4A, S2 Table), and was the group exhibiting the lowest level of IL-5 (Fig 4B, S2 Table), which translated to the highest ratio of IFN-γ to IL-5 expression (median of fold change = 17.12, *p* = 0.16, Fig 4C, S2 Table). In *PsSP52-*, *PsSP41-* and *PsSP42-*immunized mice, IL-5 expression was significantly higher compared to the control plasmid group (*p<0*.*01* in *PsSP52*, *p* = 0.01 in *PsSP41* and *p* = 0.03 in *PsSP42*, Fig 4B, S2 Table). Furthermore, neither the IFN-γ expression nor the ratio of IFN-γ/IL-5 expression in these groups was significantly different from the control plasmid group (Fig 4A and 4C, S2 Table). The data also revealed that there was no significant difference in terms of IL-5 and IFN-γ expression between the *PsSP40-*immunized mice and the control plasmid group (Fig 4A and 4B, S2 Table). The exact *p* values and median (Q1-Q3) for the six tested samples are presented in S2 and S3 Tables.

### DNA immunization with the *Ph*. *sergenti* salivary molecule *PsSP9* confers protection against *L*. *tropica* in BALB/c mice

**Fig 5:**
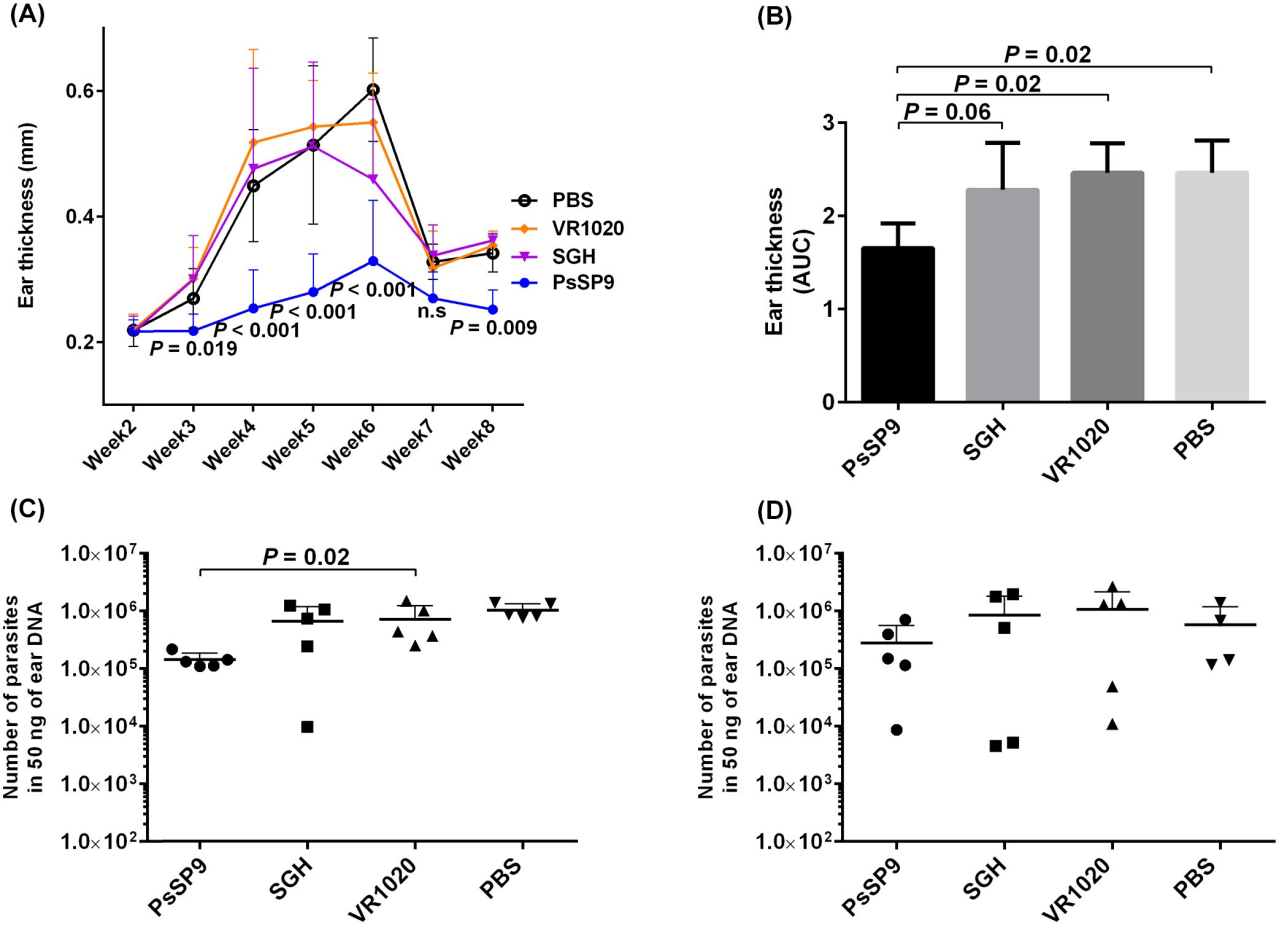
The course of infection in *PsSP9*-immunized BALB/c mice after challenge with *L*. *tropica* plus *Ph*. *sergenti* SGH. BALB/c mice (ten mice per group) were immunized in the right ear three times at two-week intervals with *Ph*. *sergenti* SGH, plasmid encoding PsSP9, empty plasmid (VR1020) or PBS. Two weeks after the last immunization, mice were challenged with metacyclic *L*. *tropica* plus *Ph*. *sergenti* SGH in the contralateral ear. Data shown are from one representative of two independent experiments. (A) The course of nodule development was monitored weekly by measuring ear thickness. Bars represent the means ± SD). (B) Disease burden was calculated as the area under the curve (AUC) obtained from (A). Ear parasite loads were determined at (C) one and (D) two months post-infection by Real-Time PCR (four to five mice per group). The *p* value in A, C and D are indicated for each vaccinated group compared with control plasmid group (VR1020) and the *p* value in B is indicated for each groups compared with PsSP9 plasmid group.

Based on the findings that the *PsSP9*-immunized group induced a Th1 immune response, we examined whether immunization with DNA coding for *PsSP9* salivary protein can lead to protection of animals against *L*. *tropica* infection. BALB/c mice were immunized intradermally in their ears three times (with two-week intervals) with either *Ph*. *sergenti* SGH, plasmid encoding a *PsSP9* salivary protein, PBS, or empty plasmid. Two weeks after the last immunization, animals were challenged with metacyclic forms of *L*. *tropica* plus *Ph*. *sergenti* SGH. *PsSP9*-immunized mice had significantly smaller nodules, indicated by ear thickness measurements, compared to the control plasmid group which showed a significantly greater ear thickness (Fig 5A, S4 and S5 Tables). The disease burden was determined based on the area under the curves (AUC) as shown in Fig 5B. The data indicated that there was a significant reduction in the disease burden after *PsSP9* immunization, in comparison with the plasmid control group (*p* = 0.02, Fig 5B, S6 Table).

One-month post-infection, the parasite load in the ear of *PsSP9*-immunized mice was significantly lower than the control plasmid group (7.2×10^5^ parasites in the control plasmid group and 1.4×10^5^ parasites in the *PsSP9*-immunized group, *p* = 0.02, Fig 5C, S7 Table). At two months post challenge, the parasite load in the ear in *PsSP9*-immunized mice remained lower than that in the plasmid control mice group, but the difference was not statistically significant (1.0×10^6^ parasites in control plasmid group and 2.7×10^5^ parasites in PsSP9-immunized group, *p =* 0.36, Fig 5D). The reduced parasite load correlated with the observed lower ear thicknesses in *PsSP9*-immunized mice. SGH-immunized mice showed no significant differences in ear thickness or disease burden compared to the control groups (Fig 5A and 5B, S4–S6 Tables). In addition, at one and two month post-infection, there were no statistically significant differences in the ear parasite burden in SGH-immunized mice compared with the control groups (Fig 5C and 5D, S7 Table).

### Protection from *L*. *tropica* infection in *PsSP9*-immunized mice correlates with increased IFN-γ expression

**Fig 6:**
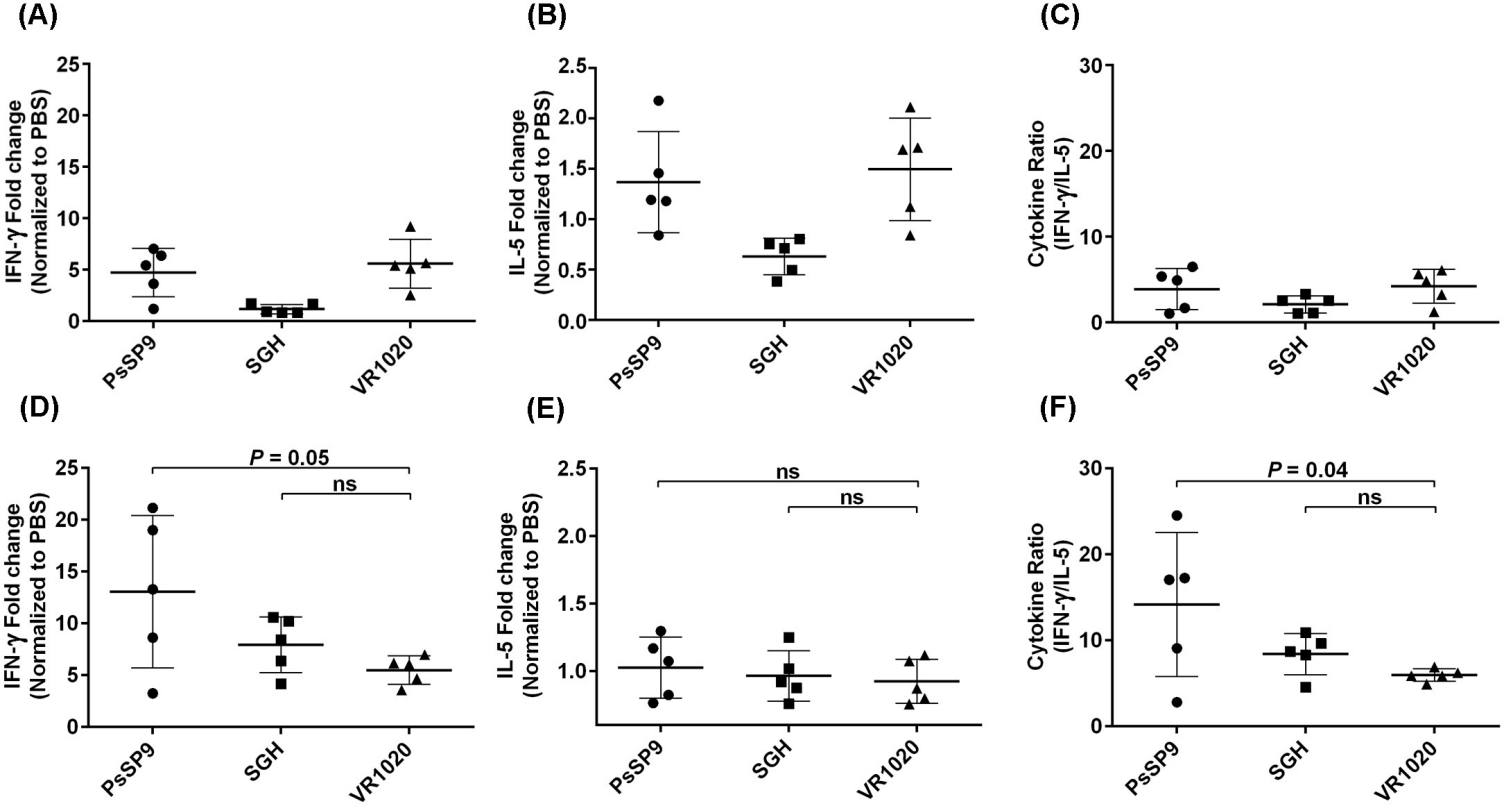
Cytokine expression profile in the dLN of *PsSP9*-immunized mice a-t one and two months after *L*. *tropica* plus *Ph*. *sergenti* SGH challenge. BALB/c mice (ten mice per group) were immunized in the right ear three times at two-week intervals with *Ph*. *sergenti* SGH, plasmid coding for PsSP9, empty plasmid (VR1020) or PBS. Two weeks after the last immunization, mice were challenged with 10^7^ metacyclic *L*. *tropica* plus *Ph*. *sergenti* SGH in the contralateral ear. Data shown are from one representative of two independent experiments. (A) IFN-γ, (B) IL-5 and (C) the ratio of IFN-γ to IL-5 expression in the dLN of BAB/c mice (four to five mice per group) immunized with PsSP9, *Ph*. *sergenti* SGH, VR1020 or PBS, at one month post-challenge with *L*. *tropica* plus *Ph*. *sergenti* SGH. The obtained results were normalized to the expression level of HPRT. These data are presented as fold change relative to the PBS control group. (D) IFN-γ, (E) IL-5 and (F) the ratio of IFN-γ to IL-5 mRNA expression in dLN of BALB/c mice (five mice per group) immunized with PsSP9, *Ph*. *sergenti* SGH, VR1020 or PBS, at two months post challenge with *L*. *tropica* plus *Ph*. *sergenti* SGH. The obtained results were normalized to the expression level of HPRT. These data are presented as fold change relative to the PBS control. The *p* value is indicated for each vaccinated group compared with the control plasmid group (VR1020).

The cytokine profile expression in the dLN was assessed at one and two months post-challenge. At one-month post-challenge, no significant differences in IFN-γ and IL-5 expression levels or in the ratio of IFN-γ to IL-5 expression were observed in any of the groups (Fig 6A–6C, S8 Table). At two months post-infection, IFN-γ expression was higher in the dLN of *PsSP9*-immunized mice than in the control plasmid group, although the difference was not statistically significant (median of fold change = 13.29, *p* = 0.05, Fig 6D, S9 Table). At this time point, the expression of IL-5 in dLN of *PsSP9-*immunized mice was not different from that in the control plasmid group (Fig 6E, S9 Table). Importantly, a significant increase in the ratio of IFN-γ to IL-5 expression was observed in *PsSP9*-immunized mice compared with the control plasmid group (median of fold change = 17.06, *p* = 0.04, Fig 6F, S9 Table). Exact *p* values and median (Q1, Q3) for the 5 studied samples are presented in S4–S9 Tables.

## Discussion

It has been previously reported that bites of *Ph*. *papatasi* [13] and *Ph*. *duboscqi* [15,24], as well as the injection of *Ph*. *papatasi* [7], *Lu*. *longipalpis* [17], and *Lu*. *whitmani* SGH [16], protected rodents against leishmaniasis, manifested by reduced lesion size and decreased parasite burden. Moreover, exposure to sand fly SGH has been reported to result in a DTH response which is associated with the enhanced production of IFN-γ and IL-12 [13,37] or a higher IFN-γ to IL-4 ratio [7]. The current study demonstrated that immunization with whole *Ph*. *sergenti* SGH did not confer protection against *L*. *tropica* infection. Indeed, the ear thickness, disease burden and parasite load in the *Ph*. *sergenti* SGH-immunized mice were similar to those of the mice in the control groups. The lack of protection in the *Ph*. *sergenti* SGH-immunized mice against infection correlates with a low ratio of IFN-γ to IL-5 production in the dLN during infection. The current results were similar to the findings of Moura *et al*., who reported that the immunization of BALB/c mice with *Lu*. *intermedia* SGH resulted in a non-protective Th2 immunity [38]. In contrast to whole SGH immunization, a single sand fly salivary protein was shown to influence the outcome of leishmaniasis. Moura *et al*. observed that DNA immunization with *Lu*. *intermedia* salivary protein Linb-11 resulted in an intense protective cell-mediated immunity against infection with *L*. *braziliensis* [23] in contrast to the whole *Lu*. *intermedia* saliva that exacerbated *L*. *braziliensis* infection [38].

The current results from our work also suggest that distinct *Ph*. *sergenti* salivary proteins induce different immune responses. Immunization with 14 plasmids coding for *Ph*. *sergenti* secreted salivary proteins resulted in the identification of 9 salivary proteins which produced either a positive DTH response, an antibody response, or both responses in BALB/c mice (Table 1). The triggering of a humoral immunity by such proteins is not necessary for anti-leishmaniasis protection. As demonstrated by Gomes *et al*., LJM19 was the only saliva protein that induced strong DTH in hamster without any detectable antibodies and DNA immunization with this plasmid protected the animal against VL [19].

Salivary proteins that result in a Th1 type cellular immune response can be considered as suitable candidate for producing an anti-leishmaniasis vaccine. In this study, the type of immune response generated by the protective plasmid *PsSp9* was characterized by a DTH response with no detectable antibody response and a significantly high ratio of IFN-γ to IL-5 expression in the dLN compared to the control group. The current study reveals that DNA immunization with the *Ph*. *sergenti* salivary protein *PsSP7* can induce an antibody response, but no detectable DTH response. Moreover, DNA immunization with *PsSP41*, *PsSP42* and *PsSP52* produced a DTH response but induced high IL-5 expression in the dLN after *Ph*. *sergenti* SGH inoculation. Therefore, DNA immunization with these proteins shifted the immune response to a Th2 type. Hence, application of such salivary proteins of *Ph*. *sergenti* (i.e. *PsSP7*, *PsSP26*, *PsSP41*, *PsSP42*, *PsSP44* and *PsSP52*) may exacerbate *L*. *tropica* infection or cause no protective impact. Interestingly, DNA immunization with *Ph*. *sergenti* salivary protein *PsSP9* (among the 14 tested *Ph*. *sergenti* salivary proteins) produced a DTH with high mononuclear infiltration in the ear, a high ratio of IFN-γ to IL-5 expression in the dLN and no detectable antibodies 48 h after *Ph*. *sergenti* SGH inoculation. Therefore, this protein directed the immunity toward a Th1 response and was chosen as a candidate for producing an experimental vaccine against *L*. *tropica*.

The mice immunized with *PsSP9* displayed a smaller ear thickness, lower disease burden, and a lower parasite load in the ear in addition to a high IFN-γ to IL-5 expression ratio in the dLN compared with the empty control plasmid group two months after challenge. The draining lymph node is a part of the immune system, where the induction of specific immunity against an antigen occurs and where effector cells migrate to the skin and contribute to protection [39]. The protection observed in the *PsSP9*-immunized mice against CL can be explained by the anti-*PsSP9* immunity at the parasite transmission site in the ear dermis, which may facilitate direct parasite killing by macrophage activation through IFN-γ. In fact, IFN-γ acts to restrict *Leishmania* growth in macrophages of mice and humans as well as the progression of leishmaniasis [40]. Vinhas *et al*. [41] demonstrated that the peripheral blood mononuclear cells (PBMC) of individuals who were subjected to uninfected *Lu*. *longipalpis* sand fly bites, exhibited IFN-γ expression after SGH stimulation. The production of IFN-γ was also associated with *L*. *chagasi* parasite killing in a macrophage-lymphocyte autologous culture [41]. This implies that after *PsSP9* DNA immunization, the effector cells (IFN-γ^+^) might have migrated to the site of *L*. *tropica* and *Ph*. *sergenti* SGH inoculation, activating the macrophages and causing parasite killing which led to smaller lesions and reduced parasite load. The presence of an immune response against *PsSP9* at the site of the parasite plus SGH injection in the dermis of the ear may act as an adjuvant to accelerate the triggering of a proper host immune response against *Leishmania*. Macrophage activation induces inflammatory responses and prevents the growth of *Leishmania* [42] by increasing the production of ROS, antigen presentation and maturation of phagosome [43]. After taking the *L*. *tropica* parasite and transporting the antigen, macrophages and Langerhans cells migrate and then differentiate into the APCs within the LNs and trigger a DTH response by CD4 expressing T cells. According to the findings of Oliveira *et al*., the immune response caused by the salivary protein induces a protective immunity against *Leishmania*. The DNA immunization of mice with *PpSP15* resulted in a Th1 immunity against *L*. *major* [20]. In the current study, it was also hypothesized that DNA immunization with *PsSP9* may act similarly. Only two critical cytokines in the dLN of immunized mice were analyzed (i.e. IFN-γ and IL-5). Further investigation of early time points in the dLN and the ear skin at the site of parasite challenge are needed to determine precisely the contribution of anti-saliva immunity in direct parasite killing and its indirect role in accelerating the induction of immunity against *Leishmania*.

It is noteworthy that as a PpSP15-like protein family member, the *PsSP9* is related to a salivary protein (14 kDa) which has no structural or sequential homology to identified proteins in humans or other organisms. *PsSP9* protein is closely similar to *PpSP12* (70% identity), *PdSP14* (69% identity), *PpSP14* (51% identity) and *PpSP15* (46% identity) salivary proteins. *PpSP15* was the first salivary protein considered as a candidate for an anti-leishmaniasis vaccine [21]. Oliveira *et al*. showed that DNA immunization of mice with *PpSP15* triggered a DTH response and induced high levels of IL-12 and IFN-γ expression 2h post exposure to *Ph*. *papatasi* bites [20,21]. This immune response was associated with the accelerated induction of an immunity and protection against *Leishmania* [20,21]. Recently, DNA immunization with *PdSP15* from *Ph*. *duboscqi* salivary protein, as a member of the *PpSP15* family, was demonstrated to protect non-human primates against *L*. *major* which was delivered through the bite of an infected sand fly [24]. Furthermore, *PdSP15* has been reported to exhibit immunogenic characteristics in humans, considering that *PdSP15* was recognized by PBMC and sera in individuals exposed to the bites of *Ph*. *duboscqi* [24]. In this regard, it is suggested that among different *Ph*. *sergenti* salivary proteins, *PsSP9* would be a better inducer of a Th1 protective immune response in a mouse model. Further investigations are still required to assess the immunogenic characteristics of *PsSP9* as a recombinant protein vaccine for mice and whether it exhibits immunogenicity in human subjects, so as to be nominated as a potential candidate or as a component of a human anti-leishmaniasis vaccine.

In summary, the current study demonstrated that while the whole *Ph*. *sergenti* SGH did not induce any protective immune response against *L*. *tropica* infection, a particular salivary protein from *Ph*. *sergenti*, *PsSP9*, induced a powerful protection against *L*. *tropica* in BALB/c mice. These findings highlight the application of specific salivary proteins from sand flies in anti-leishmaniasis vaccination approaches, which would be improved when used in combination with a *Leishmania* antigen and/or another prophylactic agent.

## Supporting information

S1 Table Median (Q1, Q3) and *p* value differences in DTH and antibody responses of different immunized groups compared with the control plasmid group (VR1020) at 48 h after *Ph*. *sergenti* inoculation. (DOCX)

S2 Table Median (Q1, Q3) and *p* value differences in IL-5, IFN-γ and ratio of IFN-γ to IL-5 mRNA expression in dLN of different immunized groups compared with the control plasmid group (VR1020) at 48 h after *Ph*. *sergenti* inoculation. (DOCX)

S3 Table Levels of IFN-γ, IL-5 and ratio of IFN-γ to IL-5 mRNA expression in dLN of different immunized groups at 48 h after *Ph*. *sergenti* inoculation. (DOCX)

S4 Table Median (Q1, Q3) and *p* value differences in the ear thickness of various immunized groups compared with the control plasmid group (VR1020) in separate time points. (DOCX)

S5 Table Median (Q1, Q3) and *p* value differences in the ear thickness of SGHs- and PBS-received groups compared with the PsSP9-immunized mice group in separate time points. (DOCX)

S6 Table Median (Q1, Q3) and *p* value differences in disease burden (area under the curves, AUC) of different immunized groups compared with the control plasmid group. (DOCX)

S7 Table Median (Q1, Q3) and *p* value differences in the parasite burden in the ear of different immunized groups compared with the control plasmid group at one and two months after *L*. *tropica* plus *Ph*. *sergenti* challenge (1MAC, 2MAC). (DOCX)

S8 Table Median (Q1, Q3) and *p* value differences in IL-5, IFN-γ and ratio of IFN-γ to IL-5 mRNA expression in dLN of different immunized groups compared with the control plasmid group at one month after *L*. *tropica* plus *Ph*. *sergenti* challenge. (DOCX)

S9 Table Median (Q1, Q3) and *p* value differences in IL-5, IFN-γ and ratio of IFN-γ to IL-5 mRNA expression in dLN of different immunized groups compared with the control plasmid group at two months after *L*. *tropica* plus *Ph*. *sergenti* challenge. (DOCX)
