# Correction: DNA plasmid coding for *Phlebotomus sergenti* salivary protein PsSP9, a member of the SP15 family of proteins, protects against *Leishmania tropica*

**DOI:** 10.1371/journal.pntd.0007585

**Published:** 2019-07-15

**Authors:** Elham Gholami, Fabiano Oliveira, Tahereh Taheri, Negar Seyed, Safoora Gharibzadeh, Nasim Gholami, Amir Mizbani, Fatemeh Zali, Sima Habibzadeh, Daniel Omid Bakhadj, Claudio Meneses, Kambiz Kamyab-Hesari, Alireza Sadeghipour, Yasaman Taslimi, Fatemeh khadir, Shaden Kamhawi, Mohammad Ali Mazlomi, Jesus G. Valenzuela, Sima Rafati

There are errors in the Funding statement. The correct Funding statement is as follows: This work was funded by Pasteur Institute of Iran (Grant no. 752), Iran National Science Foundation (ID 940007) and International Center for Genetic Engineering and Biotechnology (ICGEB, Grant no. CRP/IRN15-02) to Sima Rafati and also partially supported by the Intramural Research Program of the NIH, National Institute of Allergy and Infectious Diseases (JGV, SK, FO). The funders had no role in study design, data collection and analysis, decision to publish, or preparation and interpretation of the manuscript.
