# Expression of Concern: DNA plasmid coding for *Phlebotomus sergenti* salivary protein PsSP9, a member of the SP15 family of proteins, protects against *Leishmania tropica*

**DOI:** 10.1371/journal.pntd.0012218

**Published:** 2024-05-30

**Authors:** The PLOS Neglected Tropical Diseases Editors

Following publication and correction of this article [1, 2] concerns were raised about Fig 1. Specifically:

- The bands in Lanes 4 and 7 appear similar and when color levels are adjusted to increase contrast these lanes appear to have similar patterns within the background.
- The bands in Lanes 5 and 8 appear similar when lane 8 is horizontally flipped, and when color levels are adjusted to increase contrast there appear to be similar patterns within the background.
- The bands in Lanes 6 and 10 appear similar, and when color levels are adjusted to increase contrast these lanes appear to have similar patterns within the background.
- When levels are adjusted to visualize the background, the following lanes appear to have repeating within-lane patterns:
  ○ Lane 1, above and below the band.
  ○ Lane 2, above the band.
  ○ Lane 6, above the band
  ○ Lane 7, above the band
  ○ Lane 11, above the band
  ○ Lane 12, above the band

A corresponding author (SR) disagreed about the first and third concerns discussed above. They stated that the published data are correct for lanes 5, 6, and 10; an incorrect image was used during the preparation of lane 8; and the original blot image for lane 7 is no longer available. The *PLOS Neglected Tropical Diseases* Editors remain concerned that lanes 4 and 7 and lanes 6 and 10 appear more similar than would be expected for independent results.

Regarding the concerns in multiple lanes containing similar repeating patterns, the responding corresponding author stated these were likely caused by indentations when the strips were handled with forceps or small debris such as dust.

The original underlying data for Fig 1 and repeat experiment data were provided (S1 File).

- Based on a comparison of the raw data versus the published figure, the PLOS Editors concluded that lane 10 data appear to be duplicated in lane 6 and lane 4 data appear to be duplicated in lane 7 in the published figure.
- The raw data (original and repeat) confirmed that bands of the expected sizes were detected for each experiment (lane), but also indicated that for lanes 1, 2, 7, 8, and 14, additional bands were detected that are not visible in the published figure.
- The corresponding author confirmed that bands were removed from lanes 1 and 2 during figure preparation to focus readers’ attention on the bands of interest. In several cases, the areas of band removal corresponded to areas where there appeared to be within-lane repeated background patterns.

The experiments in Fig 1 were used to demonstrate that the plasmids used in subsequent in vivo experiments drive expression of the expected proteins in COS-7 cells. The *PLOS Neglected Tropical Diseases* Editors consider that the raw data provided in S1 File support that His-tagged proteins of the indicated sizes were expressed from the plasmids. However, we remain concerned about the integrity of the published Fig 1 regarding additional bands evident in the raw data. Therefore, the *PLOS Neglected Tropical Diseases* Editors issue this Expression of Concern.

Readers are advised to review the data in S1 File of this notice and interpret the article’s results accordingly.

## Supporting information

S1 File Original and repeat data provided in support of Fig 1. (TIF)
