# Supplementary material for: DNA plasmid coding for Phlebotomus sergenti salivary protein PsSP9, a member of the SP15 family of proteins, protects against Leishmania tropica
Source: PLoS Negl Trop Dis. 2019 Jan 11;13(1):e0007067. doi: 10.1371/journal.pntd.0007067 (PMC6345478; doi:10.1371/journal.pntd.0007067)
Supplement: S1 Table — (DOCX) [file pntd.0007067.s001.docx]

**S1 Table.** Median (Q1, Q3) and *p* value differences in DTH and Antibody responses of different immunized groups compared with the control plasmid group (VR1020) at 48 h after *Ph. sergenti* inoculation*.

| Group | DTH response | | Ab response | |
| --- | --- | --- | --- | --- |
|  | Median (Q1, Q3) | *p* value^#^ | Median (Q1, Q3) | *p* value^#^ |
| VR1020 | 0.18 (0.18, 0.18) | - | 0.08 (0.08, 0.12) | - |
| PsSP14 | 0.20 (0.19, 0.20) | 0.98 | 0.10 (0.08, 0.24) | 0.99 |
| PsSP15 | 0.18 (0.18, 0.19) | 0.98 | 0.09 (0.08, 0.13) | 0.98 |
| PsSP20 | 0.21 (0.20, 0.22) | 0.08 | 0.19 (0.15, 0.25) | 0.22 |
| PsSP26 | 0.22 (0.22, 0.23) | <0.01 | 1.53 (0.77, 2.16) | <0.01 |
| PsSP40 | 0.26 (0.22, 0.35) | <0.01 | 0.22 (0.15, 0.20) | 0.11 |
| PsSP41 | 0.22 (.020, 0.23) | 0.04 | 0.13 (0.09, 0.19) | 0.97 |
| PsSP42 | 0.21 (0.21, 0.23) | 0.03 | 0.10 (0.09, 0.15) | 0.98 |
| PsSP44 | 0.23 (0.21, 0.28) | <0.01 | 0.30 (0.17, 0.38) | 0.04 |
| PsSP52 | 0.24 (0.23, 0.25) | <0.01 | 0.19 (0.17, 0.31) | 0.20 |
| PsSP54 | 0.21 (0.19, 0.21) | 0.93 | 0.13 (0.07, 0.17) | 0.99 |
| PsSP7 | 0.19 (0.18, 0.20) | 0.98 | 0.45 (0.12, 0.62) | 0.075 |
| PsSP73 | 0.19 (0.19, 0.20) | 0.99 | 0.11 (0.08, 0.23) | 0.99 |
| PsSP9 | 0.23 (0.22, 0.25) | <0.01 | 0.16 (0.12, 0.23) | 0.97 |
| PsSP98 | 0.20 (0.19, 0.20) | 0.99 | 0.09 (0.08, 0.13) | 0.97 |
| SGHs | 0.17 (0.15, 0.20) | 0.98 | 0.40 (0.20, 0.83) | 0.01 |

*Non-parametric Van der Waerden chi-squared test for DTH response: 65.95, d.f = 15, *p* value = <0.001; for Ab response: 57.75, df = 15, *p* value = <0.001

^#^Post-hoc analysis: Pairwise comparisons using Dunn's-test for multiple tests
