# Supplementary material for: DNA plasmid coding for Phlebotomus sergenti salivary protein PsSP9, a member of the SP15 family of proteins, protects against Leishmania tropica
Source: PLoS Negl Trop Dis. 2019 Jan 11;13(1):e0007067. doi: 10.1371/journal.pntd.0007067 (PMC6345478; doi:10.1371/journal.pntd.0007067)
Supplement: S2 Table — (DOCX) [file pntd.0007067.s002.docx]

**S2 Table.** Median (Q1, Q3) and *p* value differences in IL-5, IFN-γ and ratio of IFN- γ to IL-5 mRNA expression in dLN of different immunized groups compared with the control plasmid group (VR1020) at 48 h after *Ph. sergenti* inoculation *.

| Group | **IL-5** | | **IFN-γ** | | **Ratio of IFN-γ/IL-5** | |
| --- | --- | --- | --- | --- | --- | --- |
|  | Median(Q1, Q3) | *p* value^#^ | Median(Q1, Q3) | *p* value^#^ | Median(Q1, Q3) | *p* value^#^ |
| VR1020 | 0.92 (0.77, 0.95) | - | 3.05 (2.93, 3.98) | - | 3.87 (3.82, 4.31) | - |
| PsSP40 | 1.61 (1.49, 1.67) | 0.13 | 5.60 (3.75, 9.31) | 0.98 | 3.41 (2.47, 5.79) | 0.85 |
| PsSP41 | 2.31 (1.41, 2.50) | 0.01 | 2.57 (2.13, 2.83) | 0.98 | 1.23 (0.85, 1.83) | 0.03 |
| PsSP42 | 1.78 (1.55, 1.99) | 0.03 | 3.08 (2.51, 11.06) | 0.98 | 1.75 (1.54, 4.48) | 0.36 |
| PsSP52 | 2.14 (1.64, 2.82) | <0.01 | 3.71 (3.25, 4.19) | 0.98 | 1.78 (1.49, 2.25) | 0.07 |
| PsSP9 | 1.64 (1.27, 1.99) | 0.13 | 22.30 (14.62, 32.51) | 0.04 | 17.12 (7.38, 27.58) | 0.16 |
| SGHs | 1.00 (0.79, 1.21) | 0.98 | 6.28 (3.64, 24.48) | 0.98 | 6.57 (2.99, 33.98) | 0.53 |

*Van der Waerden chi-squared test for IL-5: 23.205, d.f =6, *p* value = 0.0007; for IFN-γ: 17.838, df =6, *p* value = 0.007; for Ratio of IFN-γ/IL-5: 20.825, df =6, *p* value =0.002

^#^Post-hoc analysis: Pairwise comparisons using Dunn's-test for multiple tests
