# Supplementary material for: DNA plasmid coding for Phlebotomus sergenti salivary protein PsSP9, a member of the SP15 family of proteins, protects against Leishmania tropica
Source: PLoS Negl Trop Dis. 2019 Jan 11;13(1):e0007067. doi: 10.1371/journal.pntd.0007067 (PMC6345478; doi:10.1371/journal.pntd.0007067)
Supplement: S3 Table — (DOCX) [file pntd.0007067.s003.docx]

**S3 Table.** Levels of IFN-γ, IL-5 and ratio of IFN- γ to IL-5 mRNA expression in dLN of different immunized groups at 48 h after *Ph. sergenti* inoculation *.

| **Group** | **ID** | **IFN-ɣ** | **IL-5** | **ratio of IFN-**$\boldsymbol{ɣ}$ **to IL-5** |
| --- | --- | --- | --- | --- |
| **PsSP20** | 20_1 | 4.798733 | 2.128948 | 2.254039643 |
|  | 20_2 | 3.688824 | 1.903511 | 1.937905593 |
|  | 20_3 | 6.054038 | 1.739412 | 3.480507276 |
|  | 20_4 | 4.509336 | 2.72172 | 1.656796412 |
| **PsSP44** | 44_1 | 1.81413 | 1.351281 | 1.342525861 |
|  | 44_2 | 16.36514 | 0.754234 | 21.69767898 |
|  | 44_3 | 16.23378 | 1.039435 | 15.61788201 |
|  | 44_4 | 1.683963 | 0.982543 | 1.713882822 |
|  | 44_5 | 19.00085 | 0.748028 | 25.40125229 |
|  | 44_6 | 3.677512 | 1.433179 | 2.565981995 |
| **PsSP54** | 54_1 | 7.000202 | 0.948219 | 7.382471003 |
|  | 54_2 | 3.050816 | 0.709901 | 4.297523571 |
|  | 54_3 | 19.75668 | 0.900928 | 21.9292644 |
|  | 54_4 | 3.812072 | 1.083518 | 3.518236388 |
|  | 54_5 | 17.60419 | 0.773342 | 22.76379433 |
|  | 54_6 | 2.241511 | 1.324498 | 1.692347787 |
