# Supplementary material for: DNA plasmid coding for Phlebotomus sergenti salivary protein PsSP9, a member of the SP15 family of proteins, protects against Leishmania tropica
Source: PLoS Negl Trop Dis. 2019 Jan 11;13(1):e0007067. doi: 10.1371/journal.pntd.0007067 (PMC6345478; doi:10.1371/journal.pntd.0007067)
Supplement: S5 Table — (DOCX) [file pntd.0007067.s005.docx]

**S5 Table.** Median (Q1, Q3) and *p* value differences in the ear thickness of SGHs- and PBS-received groups compared with the PsSP9-immunized mice group in separate time points *.

|  | **Ear thickness** | | | | | | | | | | | | | |
| --- | --- | --- | --- | --- | --- | --- | --- | --- | --- | --- | --- | --- | --- | --- |
|  | **2 week after challenge (2WAC)** | | **(3WAC)** | | **(4WAC)** | | **(5WAC)** | | **(6WAC)** | | **(7WAC)** | | **(8WAC)** | |
| **Group** | Median  (Q1, Q3) | *p* value | Median  (Q1, Q3) | *p* value | Median  (Q1, Q3) | *p* value | Median  (Q1, Q3) | *p* value | Median  (Q1, Q3) | *p* value | Median  (Q1, Q3) | *p* value | Median  (Q1, Q3) | *p* value |
| **PsSP9** | 0.21  (0.20, 0.24) | - | 0.21  (0.19, 0.25) | - | 0.23  (0.19, 0.30) | - | 0.26  (0.23, 0.34) | - | 0.29  (0.24, 0.43) | - | 0.26  (0.23, 0.31) | - | 0.26  (0.22, 0.28) | - |
| **SGH** | 0.21  ( 0.19, 0.24) |  | 0.28  (0.25, 0.36) | 0.03 | 0.47  (0.34, 0.59) | >0.001 | 0.50  (0.38, 0.64) | >0.001 | 0.53  (0.32,0.56) | >0.001 | 0.35  (0.29, 0.37) | 0.02 | 0.36  ( 0.35, 0.37) | 0.002 |
| **PBS** | 0.22 (0.2, 0.24) |  | 0.25 (0.24, .031) | 0.098 | 0.45 (0.37, 0.5) | >0.001 | 0.44 (0.42, 0.64) | >0.001 | 0.62 (0.52, 0.68) | >0.001 | 0.34 (0.3, 0.35) | 0.07 | 0.34 (.032, 0.37) | 0.008 |

*To assess the effect of groups on ear thickness, we used Linear Mixed Models for repeated measure data usisng Stata (14.0) commands.
