# Supplementary material for: DNA plasmid coding for Phlebotomus sergenti salivary protein PsSP9, a member of the SP15 family of proteins, protects against Leishmania tropica
Source: PLoS Negl Trop Dis. 2019 Jan 11;13(1):e0007067. doi: 10.1371/journal.pntd.0007067 (PMC6345478; doi:10.1371/journal.pntd.0007067)
Supplement: S6 Table — (DOCX) [file pntd.0007067.s006.docx]

**S6 Table.** Median (Q1, Q3) and *p* value differences in disease burden (area under the curves, AUC) of different immunized groups compared with the control plasmid group*.

| Group | Disease Burden | |
| --- | --- | --- |
|  | Median (Q1, Q3) | *p* value^#^ |
| VR1020 | 2.56 (2.15, 2.74) | - |
| PsSP9 | 1.59 (1.58, 1.81) | 0.02 |
| SGH | 2.38 (1.90, 2.69) | 0.98 |

*Non parametric Van der Waerden chi-squared = 7.245, d.f = 2, *p* value = 0.027

^#^Post-hoc analysis: Pairwise comparisons using Dunn's-test for multiple tests
