# Supplementary material for: DNA plasmid coding for Phlebotomus sergenti salivary protein PsSP9, a member of the SP15 family of proteins, protects against Leishmania tropica
Source: PLoS Negl Trop Dis. 2019 Jan 11;13(1):e0007067. doi: 10.1371/journal.pntd.0007067 (PMC6345478; doi:10.1371/journal.pntd.0007067)
Supplement: S7 Table — (DOCX) [file pntd.0007067.s007.docx]

**S7 Table.** Median (Q1, Q3) and *p* value differences in the parasite burden in the ear of different immunized groups compared with the control plasmid group at one and two months after *L. tropica* plus *Ph. sergenti* challenge (1MAC, 2MAC)*.

| Group | Parasite Burden-1MAC | | Parasite Burden-2MAC | |
| --- | --- | --- | --- | --- |
|  | Median (Q1, Q3) | *p* value^#^ | Median (Q1, Q3) | *p* value^#^ |
| VR1020 | 443931 (372745, 1035656) | - | 1289852 (49466, 1332713) | - |
| PsSP9 | 134254 (113421, 144157) | 0.02 | 150450 (115079, 397366) | 0.36 |
| SGH | 744410 (247798, 1087160) | 0.57 | 509814 (5224, 1773740) | 0.57 |

*Nan parametric Van der Waerden chi-squared for Parasite Burden-1MAC = 5.376, d.f = 2, *p* value = 0.068; for Parasite Burden-2MAC =0.976, df =2, *p* value = 0.614

^#^Post-hoc analysis: Pairwise comparisons using Dunn's-test for multiple tests
