# Supplementary material for: DNA plasmid coding for Phlebotomus sergenti salivary protein PsSP9, a member of the SP15 family of proteins, protects against Leishmania tropica
Source: PLoS Negl Trop Dis. 2019 Jan 11;13(1):e0007067. doi: 10.1371/journal.pntd.0007067 (PMC6345478; doi:10.1371/journal.pntd.0007067)
Supplement: S8 Table — (DOCX) [file pntd.0007067.s008.docx]

**S8 Table.** Median (Q1, Q3) and *p* value differences in IL-5, IFN-γ and ratio of IFN-γ to IL-5 mRNA expression in dLN of different immunized groups compared with the control plasmid group at one month after *L. tropica* plus *Ph. sergenti* challenge*.

| Group | IL-5 | | IFN-γ | | Ratio of IFN-γ/IL-5 | |
| --- | --- | --- | --- | --- | --- | --- |
|  | Median (Q1, Q3) | *p* value^#^ | Median (Q1, Q3) | *p* value^#^ | Median (Q1, Q3) | *p* value^#^ |
| VR1020 | 1.69 (1.12, 1.71) | - | 5.41 (3.82, 7.41) | - | 4.82 (2.22, 5.84) | - |
| PsSP9 | 1.19 (1.01, 1.82) | 0.91 | 5.42 (3.61, 6.34) | 0.75 | 4.89 (1.33, 5.89) | 0.91 |
| SGH | 0.71 (0.50, 0.75) | 0.02 | 0.88 (0.80, 1.63) | <0.01 | 2.53 (1.07, 2.55) | 0.34 |

*Nan parametric Van der Waerden chi-squared for IL-5 = 9.032, d.f =2, *p* value = 0.011; for IFN-γ =7.247, df =2, *p* value = 0.027; for Ratio of IFN-γ/IL-5 =2.235, d.f =2, *p* value = 0.327

^#^Post-hoc analysis: Pairwise comparisons using Dunn's-test for multiple tests
