# Supplementary material for: DNA plasmid coding for Phlebotomus sergenti salivary protein PsSP9, a member of the SP15 family of proteins, protects against Leishmania tropica
Source: PLoS Negl Trop Dis. 2019 Jan 11;13(1):e0007067. doi: 10.1371/journal.pntd.0007067 (PMC6345478; doi:10.1371/journal.pntd.0007067)
Supplement: S9 Table — (DOCX) [file pntd.0007067.s009.docx]

**S9 Table.** Median (Q1, Q3) and *p* value differences in IL-5, IFN-γ and ratio of IFN-γ to IL-5 mRNA expression in dLN of different immunized groups compared with the control plasmid group at two months after *L. tropica* plus *Ph. sergenti* challenge*.

| Group | IL-5 | | IFN-γ | | Ratio of IFN-γ/IL-5 | |
| --- | --- | --- | --- | --- | --- | --- |
|  | Median(Q1, Q3) | *p* value^#^ | Median(Q1, Q3) | *p* value^#^ | Median(Q1, Q3) | *p* value^#^ |
| VR1020 | 0.87 (0.80, 1.08) | - | 6.05 (4.67, 6.18) | - | 5.90 (5.86, 6.25) | - |
| PsSP9 | 1.07 (0.82, 1.17) | 0.40 | 13.29 (8.65, 19.01) | 0.05 | 17.06 (9.09 , 17.27) | 0.04 |
| SGH | 0.92 (0.87, 1.02) | 0.67 | 8.43 (6.37, 10.20) | 0.22 | 8.70 (8.30, 9.64) | 0.22 |

*Nan parametric Van der Waerden chi-squared for IL-5 =0.985, d.f =2, *p* value = 0.611; for IFN-γ =3.326, df =2, *p* value = 0.190; for Ratio of IFN-γ/IL-5 =3.169, d.f =2, *p* value = 0.205

^#^Post-hoc analysis: Pairwise comparisons using Dunn's-test for multiple tests
