# Supplementary figures and images for: Expression of Concern: DNA plasmid coding for Phlebotomus sergenti salivary protein PsSP9, a member of the SP15 family of proteins, protects against Leishmania tropica
Source: PLoS Negl Trop Dis. 2024 May 30;18(5):e0012218. doi: 10.1371/journal.pntd.0012218 (PMC11139278; doi:10.1371/journal.pntd.0012218)

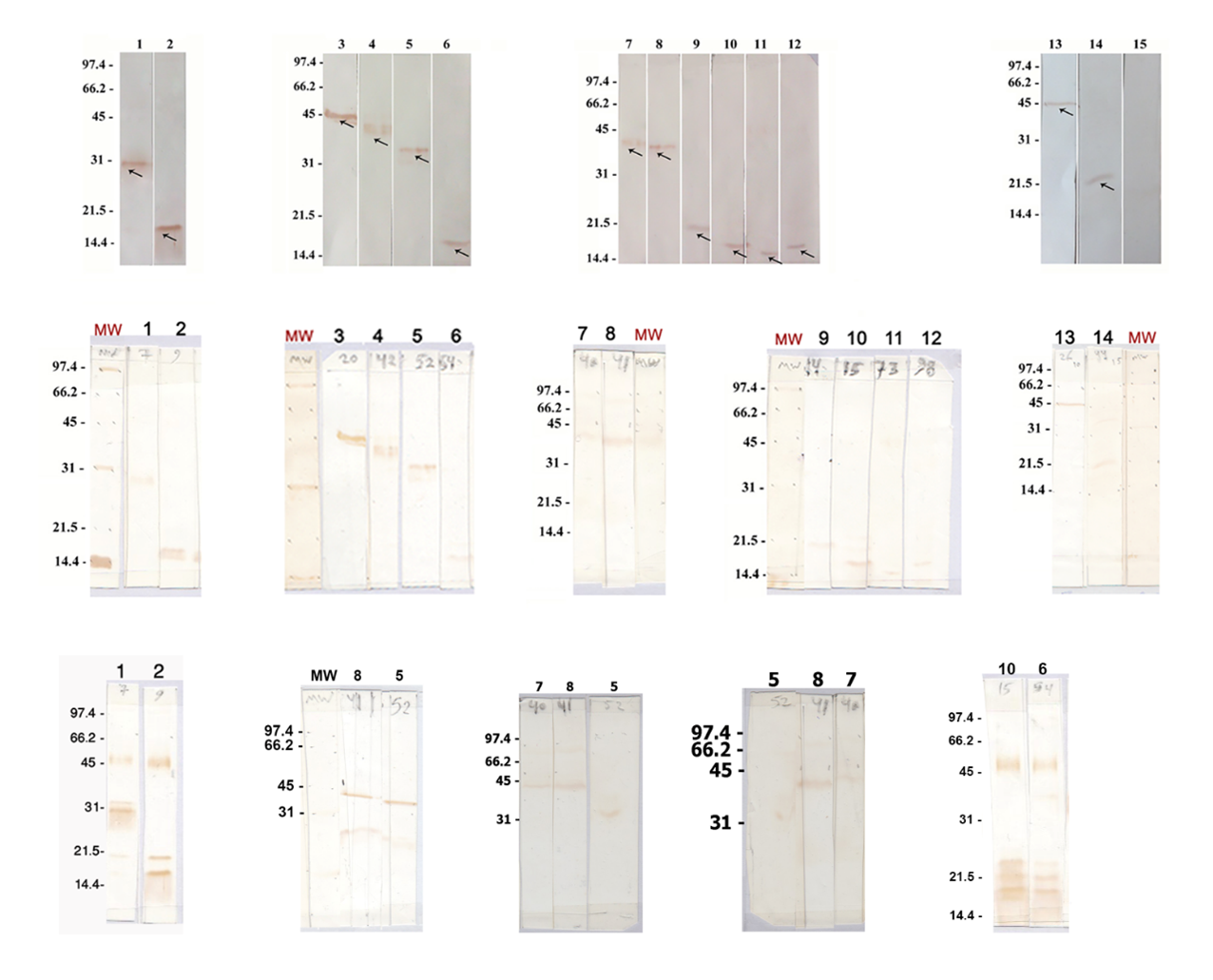

Supplement: S1 File — (TIF) [file pntd.0012218.s001.tif]
